# Introduction to high throughput synthesis, characterisation and optimisation of nanomaterials

**DOI:** 10.1039/d6na90031c

**Published:** 2026-05-13

**Authors:** Philip D. Howes, Caterina Minelli, Michael R. Thomas, Catherine S. Hansel

**Affiliations:** a Department of Engineering and Design, School of Engineering and Informatics, University of Sussex Falmer Brighton BN1 9RH UK; b National Physical Laboratory Hampton Road Teddington TW11 0LW UK; c London Centre for Nanotechnology, University College London 17-19 Gordon Street Bloomsbury London WC1H 0AH UK; d Department of Biochemical Engineering, University College London Gower Street London WC1E 6BT UK; e Generative Biology Institute, Ellison Institute of Technology The Schrödinger Building, The Oxford Science Park, Heatley Road, Littlemore Oxford OX4 4GE UK

## Abstract

Philip D. Howes, Caterina Minelli, Michael R. Thomas and Catherine S. Hansel, introduce the *Nanoscale Advances* themed issue on high throughput synthesis, characterisation and optimisation of nanomaterials.
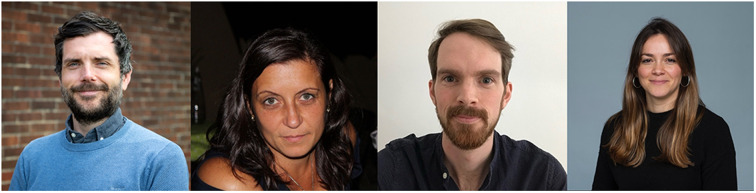

In this themed collection, we sought to gather work that bridges the gap between traditional lab-scale batch chemistry and the emerging landscape of high-throughput, automated and scalable synthesis. Bridging this gap is essential because traditional batch approaches often face bottlenecks in reproducibility, systematic parameter exploration and scaling, which ultimately constrain the pace at which new nanomaterials can be discovered, optimised and eventually manufactured and deployed. We envisioned this collection to cover both a step toward more efficient and scalable reaction pathways for nanomaterials production, and a move toward data-rich experimental strategies that lay the groundwork for AI-assisted materials design and optimisation.^1^

The discovery, development and refinement of nanomaterials have long relied on intuition and iterative manual experimentation. Today, this paradigm is being reshaped by high-throughput methodologies that integrate automation, advanced characterisation and data-driven analysis within systematic experimental workflows.^2^ These approaches enable rapid, reproducible exploration of synthesis conditions, compositional space and structure–property relationships, accelerating the translation of functional nanomaterials across diverse application areas. Modern nanoscience experiments are therefore increasingly being designed to generate expansive, quantitative datasets.^3^ As capabilities mature, we are establishing the foundations necessary for adaptive and AI-assisted decision-making in materials development.^4^ Moreover, the integration of automation, rich data capture, metadata infrastructure and continuous production systems is helping to close the gap between laboratory innovation and industrial manufacturing.

The contributions assembled in this themed collection illustrate how coordinated advances in synthesis, characterisation and optimisation are influencing applications spanning biomedicine, sensing, sustainable materials and advanced electronics. Although diverse in scope and methodology, the papers naturally cluster around three interconnected themes: the development of high-throughput experimental platforms, scalable and sustainable synthesis strategies, and data-driven characterisation and optimisation approaches. Collectively, these studies highlight an evolving movement toward accelerated and more informed discovery in nanomaterials research.

The first theme focuses on the design of automated and high-throughput experimental platforms.

In the field of therapeutic nanotechnology, Oldenburg *et al.* applied systematic high-content imaging to screen nanoparticle-based protein degraders.^5^ Their work integrated materials design with quantitative biological evaluation to link nanoparticle properties to functional performance, allowing increased understanding of structure–activity relationships. Addressing manufacturing challenges, Patil *et al.* employed a continuous coaxial-flow microfluidic reactor to generate plasmid DNA (pDNA)-loaded nanogels, sub-200 nm in diameter and with near monodispersity.^6^ The controlled flow environment enabled precise tuning of particle properties while improving reproducibility and scalability compared to traditional batch synthesis. Complementing this approach, Zimbitas *et al.* compared batch, millifluidic and continuous static mixing approaches for magnetite nanoparticle synthesis, demonstrating that continuous processing enhances production rates and enables tighter control over reaction parameters.^7^ The work describes an extensive screening of five ethylenediamine-based additives, with longer chain additives exhibiting greater morphological control. Finally, Mikami *et al.* developed a post-ageing-guided closed-loop high-throughput discovery platform.^8^ The system integrates automated synthesis, durability-focused catalytic evaluation and inverse machine-learning prediction to accelerate the design of multi-element alloy catalysts for automotive exhaust purification.

Scalability and sustainability form the second theme. Contributions here highlight how efforts in precursor and solvent screening, and more effective compositional exploration during nanoparticle preparation, can help address environmental and quality concerns that otherwise may hinder high-throughput synthesis and production.

Moreira *et al.* sought to tackle the challenge of solvent toxicity in the scale up of liquid-phase exfoliation (LPE), demonstrating that the bio-derived solvent cyrene can be used for efficient exfoliation of two-dimensional nanomaterials.^9^ Importantly, they demonstrated the efficacy of their approach on several materials, including graphene, MoS_2_, WS_2_, MoO_3_, V_2_O_5_, and hBN (hexagonal boron nitride), and the capability of producing high concentrations nanomaterial inks while reducing environmental impact. Continuing the theme of sustainability, Nava *et al.* demonstrated the conversion of waste-derived biocarbon sources (including agricultural residues and spent coffee grounds) into carbon nanodots (CNDs) through ultrafast laser ablation.^10^ This combination of sustainable feedstocks and rapid physical processing yielded a scalable and environmentally benign alternative to conventional hydrothermal or acid-oxidative syntheses.

The final theme emphasises how modern nanomaterials research increasingly relies on high-information, feedback-driven workflows that integrate synthesis, characterisation and optimisation.

Babenko *et al.* introduce a rapid crystallographic texture mapping method for atomically thin hBN films on Ni(111) by scanning electron microscopy (SEM).^11^ By leveraging secondary electron contrast, they extracted orientation information with markedly improved efficiency, lowering the barrier to large-area analysis. This high-throughput mapping capability created an efficient feedback loop for optimising growth conditions and advancing scalable manufacturing of high-quality hBN films. Additionally, Sugunan *et al.* enhanced electrochemical detection by employing liquid–liquid interfacial polymerisation of aniline on Ti_3_C_2_T_*x*_ MXene, a strategy that enabled finely controlled monomer diffusion and polymer growth to engineer uniform MXene–polymer architectures.^12^ By demonstrating how interfacial reaction environments can be tuned to dictate morphology, accessibility of active sites and electrochemical performance, the study demonstrates the power of interface-engineered synthesis within modern high-information nanomaterials optimisation workflows. Together, these works illustrate how rapid, information-rich characterisation strategies support more efficient and informed materials optimisation.

Across varied material classes and applications, the works in this themed collection demonstrate how scalable processing, sustainable chemistry and accelerated measurement can compress development timelines while strengthening fundamental understanding. Throughput alone, however, is not the ultimate objective. The greater advance lies in generating structured and reproducible datasets that inform rational design. As experimental platforms continue to mature, their integration with advanced data analytics and adaptive decision-making frameworks is likely to further accelerate discovery. This collection highlights the growing convergence of efficiency, precision and insight in the development of next-generation nanomaterials. We thank all of the authors for their contributions, and wish them all the best in their continued efforts to progress this quickly developing field of high throughput synthesis, characterisation and optimisation of nanomaterials. Finally, we thank the excellent editorial team at *Nanoscale Advances* for their assistance and continued dedication to advance the chemical sciences.
